# The Personal Health Network Mobile App for Chemotherapy Care Coordination: Qualitative Evaluation of a Randomized Clinical Trial

**DOI:** 10.2196/16527

**Published:** 2020-05-26

**Authors:** Victoria Ngo, Cynthia G Matsumoto, Jill G Joseph, Janice F Bell, Richard J Bold, Andra Davis, Sarah C Reed, Katherine K Kim

**Affiliations:** 1 Betty Irene Moore School of Nursing University of California, Davis Sacramento, CA United States; 2 Comprehensive Cancer Center University of California Davis Health Sacramento, CA United States; 3 Washington State University College of Nursing – Vancouver Vancouver, WA United States; 4 Division of Social Work California State University Sacramento Sacramento, CA United States

**Keywords:** care coordination, continuity of patient care, oncology, chemotherapy, patient-centered care, mobile health, technology adoption

## Abstract

**Background:**

Cancer care coordination addresses the fragmented and inefficient care of individuals with complex care needs. The complexity of care coordination can be aided by innovative technology. Few examples of information technology-enabled care coordination exist beyond the conventional telephone follow-up. For this study, we implemented a custom-designed app, the *Personal Health Network* (PHN)—a Health Insurance Portability and Accountability Act-compliant social network built around a patient to enable patient-centered health and health care activities in collaboration with clinicians, care team members, caregivers, and others designated by the patient. The app facilitates a care coordination intervention for patients undergoing chemotherapy.

**Objective:**

This study aimed to understand patient experiences with PHN technology and assess their perspectives on the usability and usefulness of PHNs with care coordination during chemotherapy.

**Methods:**

A two-arm randomized clinical trial was conducted to compare the PHN and care coordination with care coordination alone over a 6-month period beginning with the initiation of chemotherapy. A semistructured interview guide was constructed based on a theoretical framework of technology acceptance addressing usefulness, usability, and the context of use of the technology within the participant’s life and health care setting. All participants in the intervention arm were interviewed on completion of the study. Interviews were recorded and transcribed verbatim. A summative thematic analysis was completed for the transcribed interviews. Features of the app were also evaluated.

**Results:**

A total of 27 interviews were completed. The resulting themes included the care coordinator as a partner in care, learning while sick, comparison of other technology to make sense of the PHN, communication, learning, usability, and usefulness. Users expressed that the nurse care coordinators were beneficial to them because they helped them stay connected to the care team and answered their questions. They shared that the mobile app gave them access to the health information they were seeking. Users expressed that the mobile app would be more useful if it was fully integrated with the electronic health record.

**Conclusions:**

The findings highlight the value of care coordination from the perspectives of cancer patients undergoing chemotherapy and the important role of technology, such as the PHN, in enhancing this process by facilitating better communication and access to information regarding their illness.

## Introduction

### Background

Cancer care in the United States is fragmented [1-5] and complex [6]. The management of therapies, such as chemotherapy, requires the coordination of multiple hospital services and outpatient clinics [7]. Care coordination has been identified as a promising strategy for improving health care quality [8,9]. Engaging health care teams to actively participate in care coordination can be beneficial to patients in areas of improving communication, building trust, and facilitating transitions in care [10,11]. Successful care coordination involves effective communication among patients, their family members, and their care providers [12,13]. Communication between cancer patients and their health care team members can affect important health care decisions [14-16]. Patients face challenges such as lack of effective ways to document their health information while at the clinic or when they are away from home and lack of access care-related information [17].

It has been suggested that technology can aid in care coordination [18,19]. However, most information technology-enabled care coordination interventions have primarily utilized telephonic contact with limited examples of telehealth [9,20]. With technologies in communication and computing that have improved rapidly over the past decade, mobile health (mHealth) has enabled the collection of data, encouragement of healthy lifestyle changes, and improved management of care, especially in underserved and remote areas [21,22]. Mobile apps developed for cancer treatment can facilitate patient and provider communication, help manage patient information, and provide education around treatment follow-up [23]. Some features that may support a person’s confidence in their ability to manage their own care include calendars, logging symptoms, tracking medications, and taking notes as needed [17]. Although mHealth is promising, the specific benefits to cancer care coordination have yet to be evaluated.

### Objective

The Personal Health Network (PHN), a personalized, electronic social network built around a patient for collaboration with clinicians, care team members, caregivers, and others designated by a patient, was designed to address the challenges of cancer care coordination [24]. The objective of this study was to understand the participants’ experiences with PHN technology and to assess their perceptions of usability and usefulness of the PHN on care coordination during chemotherapy.

## Methods

### Application Description

**Figure 1:**
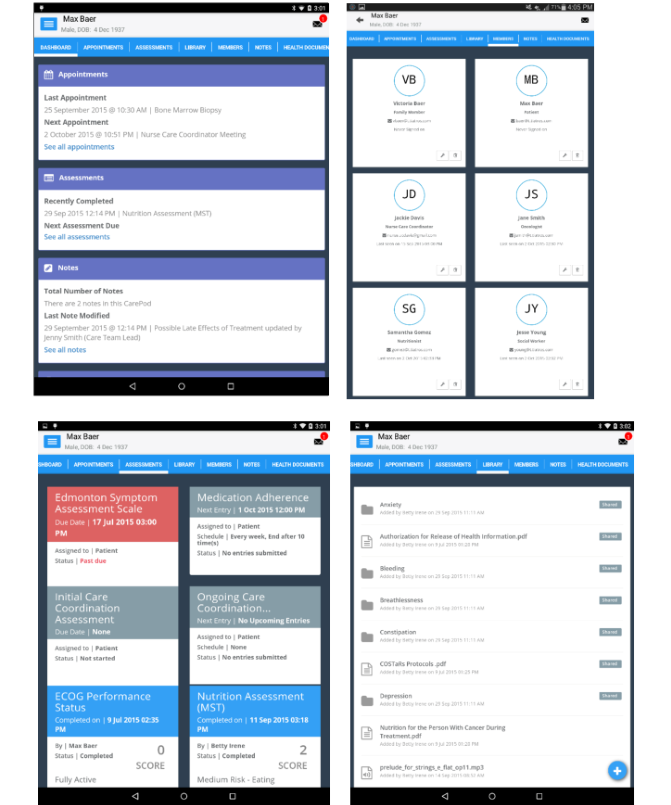
The Personal Health Network app, top left, dashboard, top right, care team including health professionals and family caregivers, bottom left, symptom assessment and person-reported outcomes, bottom right, self-management library.

The PHN care coordination mHealth app was developed by a multidisciplinary group of experts who reviewed features from the published literature, assessed prototype versions [24] and conducted a user-centered design study with an evaluation of usability among patients [25]. The app consists of a dashboard for viewing all components of the care plan, contact information for members of the care team, regular symptom assessment surveys and other validated instruments to collect data on health issues and patient-reported outcomes, a self-management library of curated health information in Web, print, and video formats, a calendar with space for open-ended notes/journaling, secure messaging, and multiparty video chat (Figure 1).

### Recruitment and Enrollment

This study was a component of a small, two-arm (N=63), randomized, pragmatic trial, in which the intervention group received the PHN technology and nurse care coordination, whereas the control group received nurse care coordination alone. Three registered nurses with care coordination training and experience provided care coordination to both arms. All trial participants were English-speaking, over the age of 18 years, received care at an urban comprehensive cancer center, had a primary diagnosis of cancer (any site), were initiating chemotherapy and had an expected survival of at least six months. All participants were followed up for 6 months after enrollment. Those in the intervention arm received an 8.4-inch Samsung Galaxy tablet with Wi-Fi and 4G data plan (Galaxy Tab Pro 8.4 SM-T325 loaded with Android 4.4 Kitkat and TouchWiz UX software), and an individual orientation to the tablet and the PHN on enrollment. Technical assistance was embedded in the PHN app, and a telephone helpline was also made available. On study completion, all participants were allowed to retain the tablet. All participants in the intervention arm were asked to participate in the interviews. This study was approved by the Institutional Review Board of the University of California Davis. Written informed consent was obtained before data collection (trial registration: NCT02238951).

### Data Collection

Interviewers used a semistructured interview guide based on concepts of the Unified Theory of Acceptance and Use of Technology [26], including usability and usefulness, impact on health, the experience of the participants using the PHN, usability of features, and barriers and facilitators to use. Interviews were conducted with the intervention-arm participants at 6 months or at the end of care coordination, whichever was earlier, recorded, and transcribed verbatim. Interviews were conducted by three individuals trained by a senior investigator who also reviewed recordings and discussed interview techniques to reinforce shared understanding and consistency among them.

### Data Analysis

A two-phased thematic analysis method [27] was utilized: first, to identify the general themes and second, to review the themes identified in the context of usability and usefulness of the PHN app. NVivo 12 Pro (QSR International) was used to organize the data during the analysis. Two analysts (VN and CG) first familiarized themselves with the data by reading through the interview transcripts. They selected words and short phrases that symbolically evoked a salient attribute (ie, single idea codes) [27,28] and noted if the terms suggested positive, neutral, or negative sentiments. The researchers collaborated on the first two transcripts to develop a draft codebook, which was used for independent coding of the remaining transcripts. Any additions or revisions to the codes were discussed and added as needed. The analysts compared their coding and worked to align the differences found. Notations were made with analytic memos for discrepancies and changes in coding. Discrepancies between coders were resolved by a third researcher (KK). The three researchers conducted a final review of all the interviews to iteratively compare and discuss the patterns that were refined into themes based on discussion and consensus.

## Results

### Participant Characteristics

A total of 33 participants were randomized to the intervention arm of the underlying trial, and 82% (27/33) participated in interviews (Table 1). One participant passed away from the disease while enrolled in the study, and the rest declined being interviewed because of scheduling conflicts. The mean age was 59 years (range 22-79 years). Most participants were female (23/27, 85%) and white (24/27, 89%). The participants were highly educated (17/27, 63% college graduates), and more than a third had high incomes (10/27, 37%, had annual incomes >US $80,000).

**Table 1:** Characteristics of interview participants.

Variables		Values, n (%)	
**Gender**			
	Male		4 (15)
	Female		23 (85)
**Age (years)**			
	18 to 45		1 (4)
	46 to 64		15 (56)
	65 and older		11 (41)
**Race and ethnicity**			
	Hispanic or Latino		0 (0)
	Non-Hispanic white		24 (89)
	Black or African American		0 (0)
	Asian		2 (7)
	Native Hawaiian or Pacific Islander		1 (4)
**Education**			
	High school graduate or GED^a^		2 (7)
	Some college		8 (30)
	College graduate		6 (22)
	More than 4-year college degree		11 (41)
**Income**			
	Less than US $49,999		7 (26)
	US $50,000 to US $79,999		9 (33)
	US $80,000 and more		10 (37)
	Prefer not to state		1 (4)

^a^GED: general education diploma.

### Thematic Analysis of the Interviews

A total of 82 single idea codes were generated and separated into positive and negative subgroups, resulting in 177 unique codes (13 codes were neutral). Key themes were uncovered through the systematic categorization of codes. In addition to overall usability and overall usefulness, 5 themes emerged from the data.

The 5 themes are listed below, and exemplar quotes are provided in Multimedia Appendix 1.

- Nurse care coordinator as a partner in care: this theme referred to someone who had a relationship with the patient, routinely checked in with the patient, helped find resources, communicated between team members, and assisted with problem solving;
- Learning: the learning theme refers to learning how to use a mobile app both via teaching and via experience using it;
- Learning while sick: participants gave insight on what it felt like to learn something new while going through chemotherapy, and for some, experiencing side effects from treatment;
- Comparison of other technology to make sense of the PHN: many participants made references to similar technologies, both apps, and devices, they used and how this knowledge was transferrable and helped them make sense of the PHN;
- Communication: the communication theme encompasses both access to people and information.

#### Overall Usability

Participants expressed that a usable app would be accessible through the internet, compatible with other apps (such as the electronic health record patient portal), easy to use, portable, navigable, and performed quickly. Some participants appreciated using the PHN because of access to information:

You know I never tried to manage my healthcare through PHN or even if I can pull MyChart into it or any of those things before. So it’s all been a new and very good experience to always have access. The accessibility is just the best part of the whole thing. It’s a 24/7 type thing.

The portability of the tablet and its ease of use allowed the participants to use the technology:

And I know that when I came here for treatment and used it, I liked using it. It was you know very small, not heavy, so it’s easy to carry with you. And like I said it’s relatively easy to use too.

Connectivity to the internet was important:

I think everybody you know I think if you’re going to push through with this program I think it’ll be very helpful for all patients as long as they have access to Internet.

Not everyone found the app easy to use or usable because of the slow performance they experienced:

And I have very, very fast internet connections and things were sluggish and kind of kludgy. I know that’s not a technological term but I was challenged and I’m looking at this thing going, this is literally driving me crazy. All this, you know, open up my laptop and go find this information quickly somewhere else.

Another participant said:

I know it’s asking a lot but that was just my initial feel because I find the other technology so easy to use and I did not find that with the one that we were using from my care for the Project.

Due to some issues with usability and software upgrades, some participants felt discouraged from using the PHN:

And I’m trying to think back over the last 6-8 months since I’ve been using it and it just seems like it’s just been really glitchy. And there has been a lot of changes so you know it seems like every time I was starting it up, I was looking at another update or another change.

And that’s sort of what it felt like for me, it’s like I mean I literally had to put on my glasses and to even see the font sizes and the buttons. And those are just navigational issues that I think that start that frustration where you go, I don’t care what else is in here because I can’t even get past the screen opening, or I can’t do those kinds of things. And I think the organization of it was confusing and it didn’t seem to make a whole lot of sense to me.

#### Overall Usefulness

Usefulness refers to the benefits of technology for participants to accomplish their health goals. Participants generally expressed that the PHN was useful because of the ability to answer questions beyond regular business hours. One person said:

It was helpful because it was on my time. So when I had to come up with a question at 7 o’clock at night when there’s no one there and come 7 o’clock in the morning when I’m really running around doing something else and maybe not having the time to sit down and think about it at that moment I was able to ask my question whenever I wanted knowing I wasn’t going to get a response till the next day but at least I wasn’t, you know I was able to deal with it then before I forgot, before something else happened. So it’s really–it’s being able to do things on my time and my schedule.

Another participant mentioned that the PHN was helpful at the beginning of the chemotherapy journey, but not so much later:

Well, when I was really sick when I was in the beginning, I used to a lot more than I of course use now because I don’t you know I’m not using it for the things that the nurse coordinator was dropping in for me and for that sort of stuff it was really, really useful.

As time progressed over a six month period I didn’t use it as much. I didn’t find it as useful because I wasn’t searching out for those answers.

Another participant wanted more interaction with the PHN:

I was hoping it to be more interactive and more personalized to me as opposed to it was kind of generic, the information that was sent.

A suggestion mentioned was that the PHN might be useful for patients who require more support:

If I was in a situation where I needed more support it would have been really well, and so I think finding out how much support does the patient want and expect. Because I had the support at home, I had other things there I didn’t feel that this was something that I needed but I can see where there are people that this would fill a gap in their care and I think it would be very well. And I’m probably not the best person to fully utilize the benefits that you have there.

### Usefulness of the Personal Health Network’s Functions

Overall, participants were more positive than negative regarding PHN functions. Table 2 shows the number of positive and negative comments made for each function. Participants identified ways in which these functions were useful to their overall health goals or specific needs related to chemotherapy care. Examples of usefulness are summarized in Multimedia Appendix 2.

**Table 2:** Perception of the Personal Health Network by function.

Function	Positive comments, n	Negative comments, n	Neutral comments, n	Total, n
Library	72	12	4	88
Survey	30	18	0	48
Messaging	34	11	0	45
Camera	1	6	0	7
Calendar	4	8	19	31
Overall interface	1	8	0	9

## Discussion

### Comparison With Prior Work

As digital technology continues to develop and create more opportunities to provide health worldwide [29], the implementation of a digital health ecosystem—where the community health network of people, devices, and technology are interconnected—must take into consideration not only the interactions of technologies but also the network and interaction of key health care stakeholders both in receiving and providing care [30,31]. Especially in digital health ecosystems within the care and assistance domain, not all stakeholders are often invited to participate in the technology design process [32]. There have been few randomized controlled trials (RCTs) of mHealth in cancer care coordination described in the literature, and fewer still that involved clinicians and patients in the design and testing of the technology [33]. Before this study, the research team proposed a conceptual framework for person-centered, community-wide care coordination and defined the concept of *point of need* for coordination, which includes both settings where health care services are delivered and everyday settings where individuals need to make health-related decisions [9,34]. In addition, a user-centered design study of the PHN prototype investigated the usability of the platform to improve the design before starting the RCT [25].

### Principal Results

The work presented in this paper investigates both usability and usefulness upon completion of care coordination among individuals undergoing chemotherapy. Similar to other studies [33,35,36], our findings show that adults find mobile apps useful for monitoring symptoms and side effects, and as a way of communicating needs and coordinating care in a timely manner [37]. Although the literature on cancer care coordination activities in the United States is sparse [38], the themes that emerged from our summative interviews contribute viewpoints that can enhance future interventions and the design and implementation of mobile apps for this purpose.

An important aspect of care coordination is access to health information. Individuals with cancer and their caregivers want information about the illness, treatment options, care needs, and often turn to the internet to seek resources [15,39-41]. Individuals with cancer face challenges in managing their health-related information [17], which includes collecting relevant data, communicating about that data, and accessing informational resources to make sense of the data. There can be barriers to access, such as paywalls or membership-only portals [39]. Moreover, individuals may experience information overload, where synthesizing information becomes an obstacle [42,43].

mHealth is well suited to facilitate information management tasks and may support health impacts [44]. The PHN, which was designed with these information management tasks in mind, was viewed as a supportive platform for accomplishing them. Although having the PHN was helpful for organizing information and care, the presence of a nurse care coordinator was important to help with problem solving. The PHN complemented the knowledge and experience of a nurse care coordinator to help guide, organize, and tailor information. The PHN library feature, in particular, received a substantial proportion of positive sentiments, indicating that it was viewed as very beneficial to participants. Participants shared that it was most helpful when the nurse care coordinator worked with the patient to identify specific information in the library and highlight it on the participant’s dashboard. It may be that the PHN library offered benefits in reducing this overload by curating relevant and clinician-endorsed information.

PHN symptom surveys were designed to increase awareness of symptoms, communicate about symptoms for early intervention, and track progress based on the patient-reported outcomes. Participants found that filling out the PHN symptom surveys was a simple task. They indicated that these surveys prompted them to think about their symptoms and discuss them with the nurse care coordinator who could deliver useful self-management information via the PHN or alert a physician for possible changes in therapy. This finding is aligned with previous studies that reported that tracking and reporting symptoms cause patients to reflect on their own well-being [45]. For those who were undergoing chemotherapy, tracking symptoms in real time increased awareness of self-care and improved communication with the health care team [46]. This also paves the way for the potential to improve clinical outcomes. Basch et al [47] reported that an intervention using Web-based symptom collection, evaluated in a large RCT among patients with cancer, was associated with improved outcomes, including quality-adjusted survival rates, fewer emergency department visits and hospitalizations, and improved quality of life. Thus, the combination of symptom awareness, self-management support, and early intervention shows promise in improving both clinical and person-centered outcomes in cancer care.

PHN messaging features were also viewed positively. Participants expressed the importance of communication outside of regular business operating hours. One participant highlighted the stress caused by not being able to communicate with a member of the health care team when experiencing an unfamiliar symptom. Although messages in the PHN were not monitored outside of business hours, there may have been some comfort in being able to express concern at the moment with the confidence that the care coordinator would respond the next morning. In our on-demand information era, consumers have become used to a very quick turnaround on questions and concerns, and this expectation has added urgency when involving a health care concern.

Although participants were willing to use the PHN, difficulties associated with learning something new while sick was a reality. Participants experiencing *chemo brain*—a term participants used to describe how they feel their thinking is impaired during chemotherapy treatment—emerged as a challenge to adopting new technology or intervention. Even though the need for information resources during initial diagnosis and early treatment may be great, the ability to adopt a tool such as the PHN may be difficult. Future study designs might target caregivers who are actively involved in care coordination. The PHN may also be an aid to survivors who are managing maintenance therapy or a survivorship care plan. In addition, participants did not use the tablet’s built-in camera, but some were enthusiastic about trying to show their doctor *something* (they found concerning on their body) or video chat face-to-face with a care team member. A future study may consider the different preferences of communication routes (eg, telephone, messaging, video conferencing, or virtual reality) when coordinating care.

Perceptions of the usability of the PHN were mixed. Participants made suggestions for improvements in navigation within the app, visual layout and increased font size and graphics, confirmation of tasks completed, and reminders for upcoming tasks. Even in the current environment of ubiquitous access to broadband, participants still reported challenges connecting to the internet (a data plan was provided). Interoperability with the patient portal in the electronic health record was highly preferred. There is room to improve technology to further enhance adoption.

### Limitations

There were several limitations to this study. Participants were recruited from one urban cancer center and were primarily older, white females with a higher socioeconomic status. Interviews were conducted as each participant completed their study period, and the sample size was determined based on an RCT. Coding was conducted after all the interviews were complete. We were not able to add interviews to assure theoretical saturation or explore new avenues of inquiry. Thus, our analysis offers limited perspectives on the usefulness and usability of PHNs.

### Conclusions

This study contributed to expanding the knowledge of cancer care coordination efforts, specifically around incorporating the use of technology to organize information, services, and people. Insight into the patient experience of PHN during chemotherapy provided a better understanding of participants’ perceptions of usability and usefulness. Findings from this analysis revealed that participants believed that care coordination is a valuable benefit for cancer patients undergoing chemotherapy, and the use of PHN technology can enhance this process by facilitating better communication and access to information.

## Appendix

Multimedia Appendix 1 Exemplar quotations for themes.

Multimedia Appendix 2 Exemplar quotations of the usefulness of the Personal Health Network features.

## Abbreviations

mHealth: mobile health
PHN: Personal Health Network
RCT: randomized controlled trial
