# Supplementary material for: The Personal Health Network Mobile App for Chemotherapy Care Coordination: Qualitative Evaluation of a Randomized Clinical Trial
Source: JMIR Mhealth Uhealth. 2020 May 26;8(5):e16527. doi: 10.2196/16527 (PMC7284410; doi:10.2196/16527)
Supplement: Multimedia Appendix 1 [file mhealth_v8i5e16527_app1.docx]

**Multimedia Appendix 1.** Exemplar quotations for themes.

| Theme | | Representative quotations |
| --- | --- | --- |
| **Nurse care coordinator as a partner in care** | | |
|  | Participants often praised their assigned nurse care coordinator, mentioning prompt attention and help received during their chemotherapy, and appreciation for their presence. | “I think it will be very good for any patient to have that care coordination as long as you know who are the people that... you’re working with. Because they’re very accessible. Because I remember when I would have a question they’ll answer me back right away. I’ll get a response right away.” |
|  | Another user who felt confident in providing care for herself said: | “I know how to take care of myself. I think (my nurse care coordinator) just reinforced, you know, you’re doing everything right. This is good. It made me feel like, okay, I’m on the right track, you know and this is normal. She was just helpful to be there because you go up and down, you know you have good days, bad days and sometimes you’re just like, God! And she would call, you know. So yeah, it was helpful, it was like she was a friend that I could talk to and not worry about you know explaining. God, I feel down. I would never want to say that to anybody, you know we’re just dragging here and I want to get over this. And, I don’t look forward to my next chemo....” |
|  | The PHN^a^ provided information and resources through the app, but the nurse care coordinator acted as a bridge between the patient and the technology | “Well, finding it on the Internet I’m–I’m not really tech savvy. So there is so much stuff and you don’t know what to go into. By talking to (my nurse care coordinator) and her finding stuff and getting it and pointing me where I could get directly to what I wanted and get the answers or the information that I needed to read and to calm me down or make me feel better or just know it was part of you know the whole journey that I’m on.” |
|  | The purpose and predictability of the nurse care coordinator calls was important | “…it was never really made really clear as to what her role was to me. I just thought she was coordinating, like if I was having any troubles with my entire care or if I was having any questions about my entire care she would direct me in that, or send me off in the direction. I never really got any clear you know definition as to what her role was except to kind of keep a cover over on me.” |
|  |  | “There were times we had calls scheduled and for reasons, legitimate reasons, she wasn’t able to call on that day and needed to call at a different time. That was a bit of an inconvenience at times.” |
| **Learning** | | |
|  | Some participants stressed the importance of receiving orientation to navigate and use the PHN | “It’s helped quite a bit because I kind of can figure out where to go, what to do and what to do when you get where you are or where you need to be to get somewhere else. Sometimes it’s a matter of just pressing the right button and see(ing) what happens. You know you just keep pressing buttons until you see a logout or a library or whatever. So it’s still there is a little bit of trial and error just because it’s a different format, that’s all.” |
|  | Supportive training measures mentioned by participants included offering additional face-to-face training in the clinic, providing written training materials, and offering a class | “I think a very short instruction manual that would have come with it, that would have said, if you need to find this, this is what you need to do, that would have been helpful for me.” |
|  | Not all participants learned the same way. A participant shared his experience on how learning may differ with age | “Well, you know I’m 67 years old and through old school where you know everything pretty much is done over the phone and in person and you get used to hopping around on a tablet and going through the different—It did give me a little bit of time on it and I tell you I’m not totally familiar with it now to get used to either the verbiage or how to actually use something. It took me forever to figure out how to send a note if you want to know the truth.” |
| **Learning while sick** | | |
|  | Some participants felt like the app would not be more helpful to people who were sicker | “It was just too much work. Because when you’re going through all of this treatment I, you know probably like other people we don’t have a lot of energy to do a lot of stuff or concentration ability. So anyway, it might be user friendly but I didn’t have the energy to deal with it.” |
|  |  | “…there is a lot of stuff going on mentally and fear and worry and confusion and new stuff... I’m nauseous and now I have to deal with something that is difficult to navigate and answer questions that really are you know kind of they’re just not pleasant questions to ask about how sick I feel.” |
| **Comparison of other technologies to make sense of the PHN** | | |
|  | Some participants who already had devices were loyal to those devices | “And plus, my phone is very similar to it so if I was out somewhere I could you know if I had that I could punch into it and see what I needed to do next or whatever.” |
|  |  | “I have my own iPad, which is similar to the tablet that they have that has allowed for me to use it.” |
|  |  | “We’re just really super loyal to the product and we’re used to thinking that way. But PC apps think kind of that way but there are some small differences that just makes us nuts.” |
|  | The ability to access the PHN from other devices was advantageous | “Because I already use the computer for work and so I would already be on the computer. And so when you’re already on it and you check your email and I’d see that I got one from or whatever, you know you can just push on the link and it takes you right to the site and I could just do the survey right there rather than like you know go to the other room, find my tablet you know get on, go to the site and take the survey on the tablet. I was already working on the computer so that’s what made that easier. And plus, the screen is a whole lot bigger.” |
|  | Interoperability of the PHN with other systems, particularly MyChart (a patient portal offered by Epic EHR^b^), was desired | “Because when you have so many appointments you don’t want to miss any of them. And so, and I still do, I log into [facility] MyChart constantly to like double check my appointments so I don’t miss any.” |
|  |  | “It was good insofar that I needed to communicate with the non-clinical side of my treatment plan. And by non-clinical I mean if I wanted to communicate with my oncologist or the nurses or the NP or something on the clinical side I still had to log into My Chart and go through that process. It would have been nice to have everything all in one.” |
|  | A few people used the device to pass the time | “I also have my games on here that I sit and play while I’m waiting for my appointments or waiting for the doctor to come in or doing the chemotherapy. I learned to do all that stuff on this, you know on this tablet.” |
| **Communication** | | |
|  | Participants used the PHN technology to find information more quickly | “I am able to access information quickly. So, for instance when I’m having a symptom like terrible digestion problems and my sister is there to help me and she says, what can I do? What can we make for you to eat? I literally did show her the document on constipation, and she read it. So, it was fast, the information was fast compared to a big binder that we got with symptom management it took more time.” |
|  | Communication using the PHN was fast and easy | “And then if I do want to make contact to any of the care coordinators and then they email me back or if I have any questions. So then in other words it’s easy for me if I want to communicate with them.” |
|  |  | “[communication should be focused] during chemotherapy, possibly every week or every other week depending on the chemo regimen because that’s when all the complications happen. That’s when people need most support.” |
|  | Although not many people shared the PHN with their family members, a few saw value in facilitating communication with family members | “I shared with my son the link to like—what’s it called?—guided imagery and he thought that was helpful so he laid down next to me and we kind of listened to it together and it was nice and relaxing.” |
|  | One participant who preferred other forms of communication said: | “...because it’s not my normal way of communicating it was not something that I flowed into. And I recognize very much the fact that many people who use social media for their main contact with people would find this extremely beneficial. For me personally, no it was not that much of a benefit.” |

^a^PHN: personal health network.

^b^EHR: electronic health record.
