# Supplementary material for: The Personal Health Network Mobile App for Chemotherapy Care Coordination: Qualitative Evaluation of a Randomized Clinical Trial
Source: JMIR Mhealth Uhealth. 2020 May 26;8(5):e16527. doi: 10.2196/16527 (PMC7284410; doi:10.2196/16527)
Supplement: Multimedia Appendix 2 [file mhealth_v8i5e16527_app2.docx]

**Multimedia Appendix 2.** Exemplar quotations of the usefulness of the Personal Health Network features**.**

| Function | Usefulness | Representative quotations |
| --- | --- | --- |
| **Library** | | |
|  | The library feature was a part of the PHN^a^ where participants could access information specific to their illness. Curated both by the patient and the members of the health care team, the library feature was the most accessed section of the PHN because patients wanted to learn about their disease, symptoms, and get advice on nutrition and meditations. The library feature was most helpful when the nurse care coordinator worked with the patient to put information into the library. Some patients expressed that there could be more information in the library. The majority of participants appreciated the tailored information delivered to the library based on their conversations with the nurse care coordinator. Some of the more tech-savvy participants, however, wanted more information than available during the time of this study. Participants accessed the library for information for their symptoms, nutrition, and meditation. | “The only thing that I can really honestly say to using this particular program was that I was suffering from a lot of fatigue and also a lot of, I don’t know, anxiety; that was it. And the nurse practitioner was able to send me over tapes through the tablet that I was able actually to do some yoga and some, I don’t know what it’s called but it’s deep thinking type stuff. And I wouldn’t have probably—I wouldn’t have gone and got tapes, I wouldn’t have gone and done anything. She was able to do those and send those over to me, these particular ones and it really helped.” |
|  |  | “I did, and [my nurse care coordinator] did introduce to me the library because I had questions about maybe it was nutrition, maybe it was some other kinds of things. I can’t remember now what she did put in my library but I liked that when I was overdone with information from our conversation that I could go back, oh yeah, I wanted to research this and she’s already put all these pamphlets into my library I could go in there and check it out at my time—on my timeframe. I liked that a lot, it did answer a lot of questions.” |
|  |  | “In the information there when I had looked up which would be the best foods for increasing when my energy was really down that I found helpful. That would have been probably the diet part would have been the one that was most effective.” |
| **Survey** | | |
|  | Participants shared that filling out the symptom surveys was simple and prompted participants to think about their symptoms. The participants would discuss the survey with the nurse care coordinator. Although the participants shared that the surveys were easy to complete, many suggested that there needed to be a prompt to notify when a survey is finished and the expectation on when they needed the surveys to be completed. | “So in other words, those surveys give me time to reflect on my illness and it gives me a perspective, I think on how I look at it.” |
|  |  | “I think the two best features were the library, but I also do feel like when I was answering the surveys it did give me information and helped me assess my symptoms to ask the right questions, not just with the nurse but when I followed up with the doctors. So those were the assessments I would fill out prior to talking with the nurse, she would remind me or I would get a reminder to fill out those forms.” |
|  |  | “I mean I think the only thing the PHN really was attempting to do was for me to use surveys off of it and again I found it difficult to find the surveys. And I think the organization of it was confusing and it didn’t seem to make a whole lot of sense to me.” |
| **Messaging** | | |
|  | The messaging feature allowed participants of the PHN to communicate through sending emails or real-time chats. Participants disclosed that communicating with the nurse care coordinator was useful with the messaging app. Participants appreciated the nurse care coordinators being able to respond appropriately and promptly to the messages sent. | “I actually recommended—I’m a social worker and I have a client that’s about to start radiation treatment. And when [RESEARCHER NAME] called I said, hey, is it still open? And she said, yeah, and I said, I’ve got somebody that is about to start radiation. So he called and I think he’s going to be meeting with them because he’s someone who doesn’t have a big support network and so could maybe really benefit from again the feeling that he–And I think his family if they buy into it then they might like that too because they work and it’s probably easier for them to send a message to somebody than it is to have to sit on the phone and call and wait for a call back and all that kind of stuff.” |
|  |  | “And it’s handy, or you can email them and say, I’m having this issue, call me or something like that at your convenience and then you’re not calling them at a bad time. I mean I always know that on Monday morning at 8 AM I can get [NURSE NAME] in her office. So I save everything up until Monday morning. But if I could email it to her on Sunday night then she could call me, because I never know what she’s doing when I call her at 8 AM.” |
|  |  | “It was good insofar that I needed to communicate with the non-clinical side of my treatment plan. And by non-clinical I mean if I wanted to communicate with my oncologist or the nurses or the NP or something on the clinical side I still had to log into My Chart and go through that process. It would have been nice to have everything all in one.” |
| **Camera (video and picture capture)** | | |
|  | The camera feature allowed the participant to take pictures or record videos as a means of being able to upload a profile picture for the PHN, documenting through photographs, or enabling video chatting. The interviews revealed that participants did not use this feature because they were not prompted to do so during the study. One participant mentioned that it would be nice to show disorders, such as a rash, to their physician with the camera. Another participant mentioned it would be nice to have a face-to-face chat with the nurse. | “I didn’t even know that existed. It didn’t jump out on me when I was scrolling through the menu and everything.” |
|  |  | “I never used the video or the photo portion but I could see how—I always thought, well if I needed to show them something or a rash or a reaction I could photograph it or even videotape it and then have ease of help without having to go into an appointment.” |
|  |  | “...I personally if I’m talking to somebody over a certain length of time I would really like to put a face, a name to a face. It makes it a lot more personal. I mean you know your nurses that answer the phone are great, [NURSE NAME] was absolutely wonderful, but it would be nice just to see her face-to-face. It makes it—it just adds to the closeness, the relationship between the nurse and the patient.” |
| **Calendar** | | |
|  | The calendar feature was not used regularly because another calendar already existed either in the patient portal for appointments or their own calendaring system. Although participants shared that the calendar system had a better feature because of the ability to write notes for the upcoming appointment, they were unwilling to use the calendar in the PHN because the patient portal offered by the hospital’s EHR^b^ had a more comprehensive view of all upcoming appointments because it was linked to the hospital’s scheduling system. Many expressed that it would be ideal if both calendars merged. | “I started initially doing my calendar in there, but I had trouble getting into the calendar. I didn’t remember. How did I get into that? You know, and I’d play back and forth and then I’d just get frustrated and put it away and keep my own calendar.” |
|  |  | “It might have been user friendly but I didn’t have the energy to look here at UC Davis MyChart, get my appointment and then type it in over onto the PHN and then go back and forth. Because I had so many appointments you know anywhere from 4 to 8 appointments a month probably between my chemotherapy and doctor’s appointments. So many appointments that I just didn’t manually want to enter all of those appointments. And sometimes they’re constantly changing too, you know the appointments. So it would be nice if the calendar system for the PHN was somehow merged with the calendar system of the UC Davis MyChart and so it overflowed.” |

^a^PHN: Personal Health Network.

^b^EHR: electronic health record.
